# Extracorporeal myoglobin removal in severe rhabdomyolysis with high cut-off membranes—intermittent dialysis achieves much greater clearances than continuous methods

**DOI:** 10.1186/s13054-021-03531-7

**Published:** 2021-03-09

**Authors:** Jakob Gubensek, Vanja Persic, Alexander Jerman, Vladimir Premru

**Affiliations:** 1 Center for Acute and Complicated Dialysis, Department of Nephrology, University Medical Center Ljubljana, Zaloska 2, 1000 Ljubljana, Slovenia; 2 Faculty of Medicine, University of Ljubljana, Vrazov trg 2, Ljubljana, Slovenia

Weidhase et al. recently published a randomized controlled trial comparing two renal replacement modalities regarding myoglobin clearance [1]. The authors found superior myoglobin clearance with a high cut-off (HCO) dialyzer running in continuous veno-venous hemodialysis (CVVHD) mode compared to a standard, high-flux dialyzer running in continuous veno-venous hemodiafiltration (CVVHDF) mode [1], which is not surprising, given the properties of HCO membranes. HCO dialyzers were first used in intermittent hemodialysis with the aim of free light chains [2] and myoglobin removal [3], but have later also been used with continuous dialysis techniques, mainly with the aim of improving clearance of inflammatory cytokines.

The authors concluded that CVVHD using HCO dialyzers could be beneficial in patients with acute kidney injury and high myoglobin levels [1]. We would like to emphasize that intermittent hemodialysis would be more suitable than continuous methods for the treatment of severe rhabdomyolysis-associated acute kidney injury (AKI). While myoglobin clearance was indeed higher in the HCO-CVVHD group, the absolute values were expectedly low, about 8–10 ml/min [1], because continuous methods have low clearances by design and cannot fully take advantage of the HCO membranes. On the other hand, using HCO dialyzers with intermittent dialysis a much greater median myoglobin clearance of 77 ml/min in hemodialysis [4] and 93 ml/min in hemodiafiltration mode [3] was reported. Although continuous methods compensate for low clearance by prolonged dialysis time, removal of clinically significant amounts of myoglobin is difficult to achieve. There is only one case report describing significant removal with high-dose continuous veno-venous hemofiltration (at 4 l/h of infusate) in a patient with very severe but transient rhabdomyolysis due to serotonin syndrome [5]. Such high-intensity continuous dialysis is quite cumbersome to perform and also costly, while intermittent dialysis can easily achieve effective myoglobin removal in 6–8-h sessions [3, 4], which can be extended to 12 h in extreme cases. The new medium cut-off membranes may prove to be an even more effective method for extracorporeal myoglobin removal because they cause less albumin loss.

In conclusion, while the role of extracorporeal myoglobin removal in severe rhabdomyolysis-associated acute kidney injury is not yet established, we suggest using HCO dialyzers with intermittent or extended dialysis techniques to achieve optimal myoglobin clearance in a cost-effective and time-efficient way.

## Data Availability

Not applicable.
